# Augmentation Mammoplasty Under Tumescent Local Anesthesia: A Multicenter Retrospective Analysis of 1644 Consecutive Cases—Safety and Efficacy in Subglandular and Submuscular Approaches

**DOI:** 10.3390/jcm15103735

**Published:** 2026-05-13

**Authors:** Emilio Trignano, Silvia Vacca, Federico Ziani, Giovanni Arrica, Sofia De Riso, Antonio Rusciani, Anna Manconi, Claudia Trignano, Corrado Rubino

**Affiliations:** 1 Plastic Surgery Unit, University Hospital Trust of Sassari, 07100 Sassari, Italy; etrignano@uniss.it (E.T.); zianifederico@gmail.com (F.Z.); giovanni.arrica@aouss.it (G.A.); sofia.deriso@aouss.it (S.D.R.); anna.manconi@aouss.it (A.M.); corubino@uniss.it (C.R.); 2 Department of Medicine, Surgery and Pharmacy, University of Sassari, 07100 Sassari, Italy; 3 Private Practice, Centro Medico Chirurgico Trignano, 07100 Sassari, Italy; 4 Private Practice, Skinlaser Center, 00196 Rome, Italy; a.rusciani@gmail.com; 5 Department of Biomedical Sciences, University of Sassari, 07100 Sassari, Italy; claudia.trignano@uniss.it

**Keywords:** tumescent local anesthesia (TLA), breast augmentation, submuscular breast augmentation, subglandular breast augmentation, hydrodissection

## Abstract

**Background**: Breast augmentation is traditionally performed under general anesthesia, but tumescent local anesthesia (TLA) offers advantages in terms of rapid recovery and reduced risks. This study presents the largest European series on the use of TLA for breast augmentation, analyzing the cumulative results of 16 years of experience. **Methods**: A multicenter retrospective analysis was conducted on 1644 consecutive patients (982 subglandular and 662 subpectoral) between 2008 and 2024. All procedures were performed under TLA with conscious sedation without the use of general anesthesia. The tumescent solution consisted of 25 mL of 2% lidocaine, 8 mEq of sodium bicarbonate, and 1 mL of epinephrine (1 mg/1 mL) in 1000 mL of 0.9% saline solution. Infiltration protocols differed between groups: the subglandular approach utilized a single-plane technique (mean 589 mL per breast), whereas the subpectoral approach required a two-stage process (pre-fascial and retromuscular) with a higher mean volume (770 mL per breast). Intraoperative parameters, complication rates, and patient-reported outcomes (BREAST-Q) were analyzed. Statistical comparisons between the two surgical planes were performed using Independent Samples T-tests. **Results**: The procedure was successfully completed under TLA in 100% of cases, with no conversions to GA. The subpectoral approach was associated with significantly higher mean operating times (141 ± 11.2 min vs. 90.3 ± 11 min; *p* < 0.001) and TLA solution volumes (770 ± 16.1 mL vs. 589 ± 53.6 mL; *p* < 0.001). The overall major complication rate was 4.74%, with a significantly higher incidence of hematoma in the subpectoral group compared to the subglandular group (3.51% vs. 1.83%; *p* = 0.015). Regarding severe capsular contracture (Baker III–IV), although a slightly higher incidence was observed in the subpectoral cohort compared to the subglandular group (2.11% vs. 1.22%), this difference was not statistically significant (*p* = 0.155). Patient satisfaction via Breast-Q was high, with dissatisfaction exclusively linked to implant dislocation. **Conclusions**: This 16-year cumulative analysis validates TLA as a safe, effective, and reproducible alternative to general anesthesia for both subglandular and subpectoral breast augmentation. While the subpectoral plane entails longer surgical times and a slightly higher risk of minor complications, the TLA protocol ensures excellent pharmacological safety and rapid functional recovery, supporting its use in modern outpatient surgical settings.

## 1. Introduction

Breast augmentation continues to be one of the most requested cosmetic surgery procedures globally [1,2]. While general anesthesia (GA) has traditionally represented the conventional approach for this surgery, it carries intrinsic risks such as postoperative nausea and vomiting (PONV), prolonged recovery times, and high costs [1,2]. In recent decades, the focus on less invasive procedures and improved postoperative management has led to the adoption and systematic use of techniques that allow for rapid discharge and immediate functional recovery.

Tumescent local anesthesia (TLA), introduced by Klein in 1987 for liposuction, has revolutionized soft tissue surgery by providing regional anesthesia, greater ease in pocket dissection and vasoconstriction with a relative reduction in pain and risk of post-operative bleeding [3,4,5].

This technique consists of the subcutaneous infiltration of high volumes of physiological solution added with lidocaine and adrenaline [6,7].

Subglandular mammoplasty has been well documented and validated as safe and effective; however, the application of this technique for submuscular placement has historically been more limited. Pectoralis major muscle dissection is considered more painful and invasive, leading many operators to prefer deep sedation or GA [1,2].

However, previous studies by our working group have demonstrated that a modified TLA protocol also allows for submuscular augmentation to be performed in complete safety, without the need for conversion to general anesthesia [1,8,9].

This study aims to present a cumulative retrospective review spanning 16 years (2008–2024), combining the experience of two surgical centers with a case series of 1644 patients.

We analyze the long-term results, safety, versatility of the technique, complications, and patient satisfaction following subglandular and subpectoral breast augmentation. All the procedures analyzed were performed exclusively under conscious sedation using TLA, demonstrating that this technique is safe and reproducible for aesthetic breast surgery.

## 2. Materials and Methods

This multicenter study was designed as a retrospective analysis of 1644 consecutive patients who underwent bilateral primary breast augmentation between 2008 and 2024. The data aggregate the historical cohorts published by Rusciani et al. [2] and Bolletta et al. [1] with the most recent consecutive data collected through 2024 at two surgical centers.

The patients were divided into two groups based on the implant placement plan: subglandular group (*n* = 982) and subpectoral group (*n* = 662).

The study was conducted in accordance with the ethical principles of the Declaration of Helsinki. All data were extracted from an integrated electronic database maintained by two surgical centers, ensuring standardized data collection across the entire cohort. The dataset includes patient demographics, intraoperative parameters, and long-term follow-up outcomes. To ensure patient confidentiality, all data were anonymized prior to analysis.

The surgical team has always consisted of a board-certified plastic surgeon, an assistant surgeon, an operating room nurse, and a board-certified anesthetist.

Patients underwent a series of preoperative tests (blood tests, cardiac tests, and ultrasound/mammography to study the breast parenchyma).

Inclusion criteria included patients with an American Society of Anesthesiologists (ASA) classification of I or II [10]. Patients with a BMI > 35, current pregnancy, or coagulation disorders were excluded. All patients provided written informed consent, understanding the nature of the surgical and anesthesiological protocols.

Both round and anatomical implants were used for the procedures described (Polytech: Polytech Health & Aesthetics GmbH, Dieburg, Germany; Silimed: Silimed Indústria de Implantes Ltda, Rio de Janeiro, Brazil; GC Aesthetic: GC Aesthetics Ltd, Dublin, Ireland; Motiva: Motiva European Distribution Center, Wommelgem, Belgium).

Before surgery, patients assessed the volume of the prosthetic implants together with the operating surgeon by wearing sizers inserted into a bra. Patients with a pinch test < 2 were not candidates for subglandular breast augmentation.

Patients stopped taking medications that interfere with coagulation 5–7 days before the surgical procedure.

Preoperative drawings were performed and photographic documentation was made.

All procedures were performed under day-surgery with the constant presence of a certified anesthetist.

In the operating room, each patient was placed with peripheral venous access and vital signs were monitored.

Sedation was achieved exclusively with intravenous Midazolam (0.05 mg/kg as premedication, with possible intraoperative boluses), avoiding the use of Propofol, Ketamine or major opioids to keep the patient cooperative and reduce the risk of respiratory depression and emesis [1,2].

The skin incision was preliminarily infiltrated in both approaches (subglandular and submuscular) with Lidocaine 1% with Epinephrine 1:100,000.

The tumescent solution (Klein formula) was prepared by adding to 1000 mL of physiological solution (NaCl 0.9%): 25 mL of Lidocaine 2% (500 mg), 1 mL of Epinephrine (1 mg/1 mL), 8 mEq of sodium bicarbonate.

The mean volume of infiltrated TLA and the specific technique were adapted to the surgical plan.

In subglandular placement, in order to correctly identify the dissection space between the parenchyma and the superficial fascia of the pectoralis major, the surgeon performed a pinch test of the breast relative to the chest wall. Once the plane was identified, a spinal needle (18G, Quinke tip) was inserted, connected to a peristaltic infusion pump, through which 400 to 700 mL of TLA were infiltrated on each side. The infiltration was suspended once evident tissue turgor and vasoconstriction were achieved. To ensure the pharmacological efficacy of the lidocaine and epinephrine, a waiting period of 45 min was observed before starting the surgical procedure.

Anesthesia support included the intravenous administration of 1 g of Midazolam five minutes before the incision. In the final phase of the procedure, approximately thirty minutes before the end of the procedure, 1 g of Paracetamol was infused IV for postoperative pain control. A 5 cm skin incision was made along the preoperative design. The dissection extended deep to the superficial fascia of the pectoralis major muscle, proceeding to create the subglandular pocket following the perimeter of the gland and adapting it to the specific dimensions of the chosen implants. In order to achieve dissection unipolar needlepoint radiofrequency forceps and Metzenbaum scissors were used, combined with a progressive coagulation of the vessels. The use of a fiber-optic retractor with an integrated smoke evacuation system allowed for an excellent view of the surgical field.

In subpectoral placement too, the operating surgeon identified the space between the gland and the fascia of the pectoralis major muscle using a pinch test. A spinal needle connected to a peristaltic pump was inserted into this plane, suspending the infusion once tissue turgor and vasoconstriction were achieved (equivalent to an average volume of 530 mL per breast). The anesthetic effect was ensured by direct contact of the solution with the tissues, respecting a 40 min latency period before the incision to optimize the effect of the adrenaline and lidocaine.

Skin access was performed with a 4 cm incision for round implants and a 6 cm incision for anatomical implants. Once the pectoralis major fascia was exposed, it was infiltrated with 1 mL of lidocaine and epinephrine. Subsequently, a cannula (2 mm × 200 mm) was inserted into the muscle body, secured with a 4–0 silk “round block” suture, through which 190–250 mL of tumescent solution was injected using a Luer-lock syringe. The total volume of infiltrated solution was between 700 and 800 mL per side, with variations calibrated based on the patient’s BMI and breast size in order to avoid reaching pharmacological toxicity thresholds. The dissection continued with electrocautery, proceeding with the inferior and medial incision of the pectoralis major muscle up to the upper limit of the areola (corresponding to the fourth-sixth rib). During pocket preparation, the use of a fiber-optic retractor with a smoke evacuation system ensured optimal visualization of the field and progressive and accurate hemostasis, a fundamental step in preventing late post-vasoconstriction bleeding.

Following strict sterility standards, surgical gloves and drapes were changed before the prosthesis was placed, and the instruments were cleaned with chlorhexidine. The pocket was then irrigated sequentially with a diluted solution of 50% hydrogen peroxide, saline solution and, finally, antibiotic solution (gentamicin/rifampin).

The procedure was completed without the use of drains, performing a layered suture with absorbable material and applying a sterile dressing.

Patients were discharged after a 2 to 4 h observation period, with instructions to wear a compression sports bra for the first 4–6 weeks. The post-discharge pharmacological protocol included a 5-day course of antibiotic therapy (amoxicillin/clavulanic acid or ciprofloxacin in case of allergy) and mild analgesics as needed [1,2].

**Figure 1:**
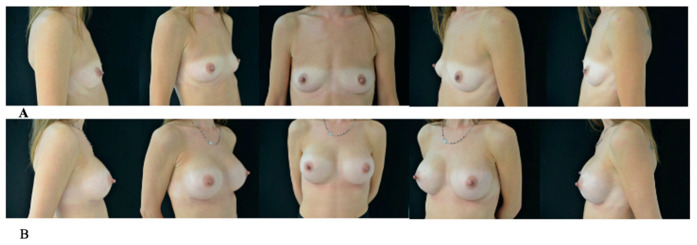
(**A**) Preoperative view. (**B**) Postoperative view after 1 year.

**Figure 2:**
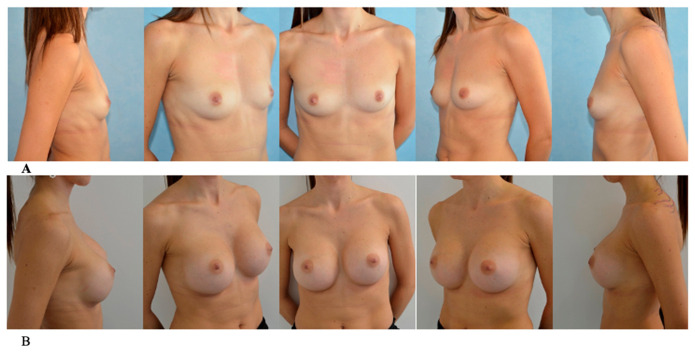
(**A**) Preoperative view. (**B**) Postoperative view after 1 year.

The check-ups were scheduled at regular intervals: 24 h after surgery, 1 and 2 weeks, and subsequently at 1, 3, 6 months and 1 year (Figure 1 and Figure 2).

## 3. Results

Between 2008 and 2024, a total of 1644 surgical procedures performed under TLA were analyzed and stratified into two groups based on the anatomical plane of implant placement: the subglandular group (*n* = 982) and the subpectoral group (*n* = 662). The surgical data are reported in Table 1. The study population presented a mean age of 35.8 years (SD ± 9.6; range 18–68) for the subglandular cohort and 36.9 years (SD ± 9.5; range 18–67) for the subpectoral cohort. Average patient weight was comparable between groups, recorded at 61.5 kg and 60.0 kg, respectively.

Regarding intraoperative pharmacological and surgical parameters, the mean Lidocaine dosage was 10.6 mg/kg in the subglandular group compared to 11.5 mg/kg in the subpectoral group. Significant differences were observed in the volume of tumescent local anesthesia (TLA) solution administered, with a mean of 589 mL per breast (range 420–700) for subglandular dissections and 770 mL per breast (range 720–800) for subpectoral procedures. The average implant volume was 329 cc for the subglandular group and 338 cc for the subpectoral group.

Procedural efficiency and postoperative management were also documented: the subglandular group showed a mean operating time of 90.3 min and a recovery time of 125 min. In contrast, the subpectoral group required a longer mean operating time of 141 min, with an average recovery stay of 150 min.

The comparative analysis of perioperative data between the two surgical cohorts revealed highly significant differences (*p* < 0.001) for all monitored continuous variables. Specifically, the Welch’s t-test (Table 2) demonstrated that the subpectoral approach required a significantly longer mean operating time (141 ± 11.2 min) compared to the subglandular approach (90.3 ± 11.0 min; t(1405) = −89.8, *p* < 0.001). Similarly, the volume of tumescent local anesthesia (TLA) solution administered was significantly higher in the subpectoral group (770 ± 16.1 mL) than in the subglandular group (589 ± 53.6 mL; t(1229) = −99.6, *p* < 0.001). Postoperative recovery times were also significantly extended for the subpectoral cohort, with an average stay of 150 ± 14.0 min compared to 125 ± 8.97 min for subglandular patients (t(1024) = −40.7, *p* < 0.001). Despite these differences, pharmacological safety was maintained in both groups, with mean lidocaine dosages of 10.6 ± 1.25 mg/kg for subglandular and 11.5 ± 1.48 mg/kg for subpectoral procedures.

Regarding the shape of the implants, 1062 surgical procedures were performed with round implants (64.6% of total procedures) and 582 surgical procedures with anatomical implants (35.4% of total procedures) (Table 3).

No cases of intraoperative conversion to general anesthesia occurred, nor were there any clinical signs of lidocaine toxicity or epinephrine-related cardiovascular adverse effects recorded.

No serious postoperative complications such as deep vein thrombosis, pulmonary embolism, or pneumothorax occurred.

Postoperative complications were recorded as dichotomous variables and analyzed for both surgical cohorts (Table 4). The adverse events monitored included hematoma, seroma, implant dislocation, and dystrophic scarring. In the subglandular group (*n* = 982), the incidence of complications was as follows: hematoma was observed in 1.83% ± 0.13 (*n* = 18) of cases, seroma in 1.73% ± 0.13 (*n* = 17), implant dislocation in 1.22% ± 0.11 (*n* = 12), and dystrophic scarring in 2.65% ± 0.16 (*n* = 26).

For the subpectoral cohort (*n* = 662), higher incidence rates were recorded across all categories: hematoma occurred in 3.51% ± 0.18 of patients (*n* = 25), seroma in 2.53% ± 0.16 (*n* = 18), implant dislocation in 2.95% ± 0.17 (*n* = 21), and dystrophic scarring in 4.21% ± 0.20 (*n* = 30). All data are presented as mean incidence rates (proportions), total event counts (sum), and standard deviations (SD) to account for statistical variability within the two anatomical planes.

The occurrence and severity of capsular contracture were assessed and categorized according to the Baker classification [11] (Table 5). For statistical purposes, each grade was recorded as a dichotomous variable. In the subglandular group (*n* = 982), the distribution was as follows: Grade I (*n* = 694; SD = 0.455), Grade II (*n* = 276; SD = 0.450), and Grades III–IV (*n* = 12; SD = 0.110). In the subpectoral cohort (*n* = 662), 493 cases were classified as Grade I (SD = 0.462), 155 as Grade II (SD = 0.413), and 14 as Grades III–IV (SD = 0.139). These data were utilized to compare the long-term fibrotic response between the two anatomical planes. Results are presented as total counts (sum) and standard deviations (SD).

Regarding severe capsular contracture (Baker III–IV), the incidence was 1.22% (*n* = 12) in the subglandular group and 2.11% (*n* = 14) in the subpectoral cohort (Table 6). A formal statistical comparison (Chi-square test) revealed no significant difference between the two anatomical planes (χ^2^ = 2.03, df = 1, *p* = 0.155) (Table 7), demonstrating that the implant placement plane under TLA did not significantly impact the rate of severe fibrotic response.

One year postoperatively, patient satisfaction and patient-reported outcomes were quantitatively assessed using the BREAST-Q questionnaire. The cohort reported high mean scores across all domains (Table 8): Satisfaction with breasts (93.2 ± 6.4), Satisfaction with outcome (95.7 ± 5.11), Psychosocial well-being (91.5 ± 6.21), Sexual well-being (88.6 ± 7.62), and Physical well-being (94.5 ± 4.58). Satisfaction with information received was the highest-rated domain (96.8 ± 2.67). As previously observed, lower satisfaction levels were exclusively associated with specific postoperative complications, such as implant dislocation. The patients in this study had a follow-up of up to 7 years.

## 4. Discussion

This multicenter retrospective study analyzes a consecutive cohort of 1644 patients undergoing surgery between 2008 and 2024, representing the largest European experience on the exclusive use of tumescent local anesthesia (TLA) in breast augmentation. The integration of the historical case series by Rusciani et al. [2] and Bolletta et al. [1] with more recent data from two centers demonstrates that standardizing the protocol allows us to overcome the historical limitations of the technique. While in the past, TLA was confined to liposuction or superficial procedures, our data confirm its applicability and safety even in complex surgical planes such as the subglandular (982 cases) and subpectoral (662 cases), traditionally domain of GA.

Safety is the cornerstone of any day-surgery surgical protocol. Our results, characterized by the absence of mortality and major systemic complications, are authoritatively confirmed in the systematic review by Liu et al. [12], which analyzed over 71,000 surgical procedures performed using TLA. Liu et al. highlight a key historical fact: while in the pioneering phase of the technique (pre-2003) deaths related to non-standardized dosages during massive liposuctions had occurred, no TLA-related deaths have been reported in the 33,429 cases published since 2003. This “zero mortality” data in the modern literature, combined with our series of 1644 consecutive cases without major adverse events, demonstrates that TLA is a safe technique.

Our data align with what was reported by Tanaka et al. [13], who recorded a 98% satisfaction rate and the absence of major systemic complications on a case series of 818 Asian patients treated with TLA. Similarly, our experience confirms that the use of TLA dramatically reduces intraoperative bleeding. Klein [14] explains this phenomenon through the vasoconstriction induced by capillary diffuse epinephrine, which allows an almost bloodless fat aspiration (or dissection, in our case). This hemostatic advantage is crucial: as also observed by Tanaka et al., vasoconstriction of the perforating branches of the internal thoracic vessels reduces bleeding to less than 5 mL per side, eliminating the need for surgical drainage in almost all cases and potentially reducing the risk of hematoma-related capsular contracture.

At the pharmacokinetic level, the safety of lidocaine at high volumes is explained by the study by Riff et al. [15]. They demonstrated that in breast tissue, which is less vascularized than other areas [8,9], the plasma peak (Cmax) of lidocaine is reached very slowly at 5.7 h and settles at average values of 1.62 µg/mL, well below the toxicity threshold (6–7 µg/mL). This slow release, due to the lipophilicity of the drug and the vasoconstriction induced by epinephrine, ensures that the peak concentration occurs when the patient is already discharged or in an advanced recovery phase, providing a protective analgesic “tail” [4].

The concept of analgesic tail in the postoperative period, also highlighted in the study by Głowacka et al. [16], is correlated with a reduced need for narcotics and a general improvement in patient comfort in the hours following the operation, as also confirmed in our study where discharge occurs in total safety within a few hours.

However, as suggested by Uttamani et al. [17], safety strictly depends on the pharmacological history to exclude cytochrome P450 inhibitors and on the immediate availability of lipid emulsion in the operating room to treat any very rare systemic toxicities (LAST) [18].

A crucial technical advantage that emerged from our experience, especially in the 982 cases of subglandular positioning and the 662 submuscular ones, is the effectiveness of hydrodissection.

As highlighted by Asal et al. [19] in comparing the tumescent technique with electrocautery for mastectomy, pressurized infiltration acts as a liquid scalpel. The solution creates bloodless anatomical planes separating structures (such as Cooper’s ligaments or the retromuscular space) before the incision, reducing the need for thermal dissection. Asal et al. report that extensive use of electrocautery increases thermal damage, the rate of flap necrosis, and the formation of postoperative seromas. Complementing these atraumatic principles, Kim et al. [18] analyzed Asian patient cohorts and noted that performing precise blunt dissection with fingers under tumescent infiltration yields highly satisfactory aesthetic outcomes equivalent to traditional electrocautery, provided that adequate single antibiotic breast irrigation is applied to prevent subsequent capsular contracture. Our data show a low rate of hematomas (2.62% in the entire population (*n* = 1644); 3.8% in the submuscular cohort (*n* = 662) and 1.8% in the subglandular cohort (*n* = 982)) and also a low-rate seromas (2.13% in the entire population (*n* = 1644); 2.7% in the submuscular cohort (*n* = 662) and 1.7% in the subglandular cohort (*n* = 982)), confirming that preventive chemical (epinephrine) and mechanical (tumescence) hemostasis are superior to reactive hemostasis.

Our case series demonstrates the versatility and applicability of TLA on different planes. Rusciani et al. initially validated the technique for the subglandular plane, and Bolletta et al. subsequently validated the technique for submuscular placement, confirming that hydrodissection allows for the safe management of even complex muscle disinsertion with patient comfort. Although muscle dissection was historically considered painful, tumescent local anesthesia (TLA) has proven adaptable to different tissue densities—including gland, fat, and muscle—while maintaining an exceptional safety profile.

This versatility is further supported by Kim et al. [20], who described the effectiveness of blunt finger dissection under tumescent infiltration for submuscular and dual-plane breast pockets, achieving reoperation rates comparable to traditional techniques.

Recent literature also details the safe use of TLA for reduction mammaplasty in large cohorts [21]. Furthermore, the specific efficacy of TLA has been highlighted in transgender patients undergoing primary submuscular breast augmentation, enabling rapid recovery and addressing gender dysphoria with minimal morbidity [22]. Similarly, Ziani et al. [23] safely utilized TLA for augmentation mastopexy, adeptly managing the structural complexities of both lifting and augmentation in a fully awake setting without major complications.

The strategic validity of utilizing TLA is powerfully reinforced by its successful application in highly complex, multi-step procedures such as Hybrid Breast Augmentation (HBA).

Trignano et al. [24] demonstrated that HBA, which combines subfascial implant placement with autologous fat grafting (AFG) to ensure optimal tissue coverage, can be executed entirely and safely under TLA. In their series, the standardized TLA solution was utilized to anesthetize both the donor sites for fat harvesting and the breast pockets. This proves that TLA is an exceptional surgical strategy: it provides a virtually bloodless field that protects graft viability during harvesting, and it allows for precise, dual-layer injection (suprafascial and subcutaneous) around the mammary gland without the need for general anesthesia.

The applicability of tumescent local anesthesia is not limited to breast surgery. Although the present study focuses on implant-based augmentation, its effectiveness has also been demonstrated in procedures involving different anatomical regions and tissue characteristics, such as medial thigh lift [25], where variable infiltration volumes and deep dissection can be safely managed while maintaining adequate analgesia and hemostasis.

Intraoperative pain management is a controversial topic. In the literature, Ditlev et al. [26] propose the routine use of intercostal nerve blocks (II–VII intercostal spaces) combined with light sedation for breast augmentation. Although effective, Ditlev reports a pneumothorax rate of 0.6% in a population of 335 patients, an intrinsic risk of performing deep blocks. Furthermore, a large Canadian advisory classifies the intercostal block as a procedure with a risk of bleeding: the vascular-nerve bundle is superficial but any hemorrhage can flow into the thoracic cavity [27]. Our layered direct tumescent infiltration technique (first subglandular, then subpectoral), initially validated by Bolletta et al. [1], proved sufficient to ensure complete analgesia in 1644 cases without incurring the risk of pneumothorax associated with nerve blocks. Another point of disagreement concerns the temperature of the solution. Some authors [28] support the use of cold tumescent anesthesia (4 °C solution) to enhance vasoconstriction and immediate analgesia. However, our experience is in line with that reported by Uttamani et al. [17]. and Joukhadar et al. [29], who report that the injection of cold fluids can significantly increase the pain perceived during infiltration and cause shivering that disrupts monitoring and patient comfort. In our protocol, the use of room temperature solution buffered with bicarbonate (to neutralize the acidity of lidocaine) proved to be the ideal compromise to avoid injection burning and thermal stress, while maintaining excellent hemostatic efficacy. Eliminating general anesthesia fits perfectly into modern ERAS (*Enhanced Recovery After Surgery*) protocols, as highlighted by Boeer et al. [30]. By avoiding major opioids and anesthetic gases, we drastically reduce the incidence of nausea and vomiting (PONV), common complications of GA [10], allowing immediate resumption of feeding and ambulation. This factor, combined with the absence of drains in almost all cases, allowed for an average discharge within 2–4 h, transforming breast augmentation (even submuscular) into a true low-impact *day-surgery* procedure and reducing the risk of venous thromboembolism (DVT) associated with immobilization [31,32]. Despite excellent results, the technique presents challenges. As noted by Uttamani et al. [17], the learning curve for the surgeon is steep: the infiltration must be precise and requires patience (20–45 min of latency for the epinephrine to take effect). An error in the injection plan can make the dissection painful or alter anatomical landmarks due to tissue turgor. Furthermore, the technique requires psychological management of the awake patient, which not all surgeons are accustomed to.

## 5. Conclusions and Limitations

In conclusion, the cumulative analysis of 1644 cases confirms that tumescent local anesthesia (TLA) represents a safe and highly effective alternative for primary breast augmentation. Our findings demonstrate that TLA provides significant clinical benefits, including reduced intraoperative bleeding through hydrodissection, a favorable pharmacological safety profile, and immediate functional recovery. Furthermore, the technique proves versatile across various implant placement planes-subglandular and subpectoral-while maintaining high levels of patient safety. The breadth of this extensive case series validates the use of TLA beyond superficial procedures, establishing it as a reliable and reproducible surgical approach for elective breast augmentation in an outpatient or day-surgery setting.

Despite the clinical significance and the large sample size of this study, several limitations must be acknowledged. First, this large multicenter series provides real-world data on the use of tumescent local anesthesia in elective breast augmentation. As with most studies in aesthetic surgery, the retrospective design and the inclusion of predominantly ASA I–II patients require that the findings be interpreted within this clinical context.

Second, the absence of a direct control group undergoing general anesthesia (GA) precludes formal comparative analysis. Finally, while all patients completed a minimum one-year follow-up, the overall duration of follow-up was variable, extending up to seven years for only a portion of the cohort. This variation may affect the long-term assessment of late complications, such as capsular contracture. Future prospective, randomized trials are warranted to further validate these findings against conventional anesthetic techniques.

## Figures and Tables

**Table 1:** Operating data in 1644 cases.

	Anatomical Plane	Age, y	Weight, kg	Lidocaine, mg/kg	Implant Volume	TLA Solution, mL	Operating Time, min	Recovery Time, min
Mean	Subglandular	35.8	61.5	10.6	329	589	90.3	125
	Subpectoral	36.9	60.0	11.5	338	770	141	150
Standard deviation	Subglandular	9.61	7.27	1.25	100	53.6	11.0	8.97
	Subpectoral	9.56	6.05	1.48	91.7	16.1	11.2	14.0
Minimum	Subglandular	18	46.8	9	181	420	70	105
	Subpectoral	18	46.4	9	181	720	105	120
Maximum	Subglandular	68	82.0	14	721	700	115	145
	Subpectoral	67	76.7	16	642	800	160	190

**Table 2:** Independent Samples T-Test.

		Statistic	df	*p*
Operating time, min	Welch’s t	−89.8	1405	<0.001
Recovery time, min	Welch’s t	−40.7	1024	<0.001
TLA solution, mL	Welch’s t	−99.6	1229	<0.001

Note: H_a_ μ_Subglandular_ ≠ μ_Subpectoral_.

**Table 3:** Number and percentage of implants based on shape.

	Round Implant	Anatomical Implant
**Sum**	1062	582
**%**	64.59%	35.4%

**Table 4:** Complications.

	Anatomical Plane	Hematoma	Seroma	Implant Dislocation	Distrophic Scar
Mean	Subglandular	0.0183	0.0173	0.0122	0.0265
	Subpectoral	0.0351	0.0253	0.0295	0.0421
Sum	Subglandular	18	17	12	26
	Subpectoral	25	18	21	30
Standard deviation	Subglandular	0.134	0.130	0.110	0.161
	Subpectoral	0.184	0.157	0.169	0.201

**Table 5:** Capsular contracture (Baker Classification).

	Anatomical Plane	Capsular Contracture Grade I	Capsular Contracture Grade II	Capsular Contracture Grade III–IV
Sum	Subglandular	694	276	12
	Subpectoral	493	155	14
Standard deviation	Subglandular	0.455	0.450	0.110
	Subpectoral	0.462	0.413	0.139

**Table 6:** Severe capsular contracture (Baker III–IV) by anatomical plane.

	Capsular Contracture III–IV		
Anatomical Plane	Yes	No	Total
Subglandular	12	970	982
Subpectoral	14	648	662
Total	26	1618	1644

**Table 7:** Chi-square test results for severe capsular contracture (Baker III–IV) by anatomical plane.

χ^2^ Tests			
	Value	df	*p*
χ^2^	2.03	1	0.155
N	1644		

**Table 8:** Breast-Q patient-reported outcomes at 1-year follow-up.

Breast-Q						
	Satisfaction with Breasts	Satisfaction with Outcome	Psychosocial Well-Being	Sexual Well-Being	Physical Well-Being	Satisfaction with Information
Mean	93.2	95.7	91.5	88.6	94.5	96.8
Standard deviation	6.4	5.11	6.21	7.62	4.58	2.67
Minimum	53	57	62	58	70	89
Maximum	100	100	100	100	100	100

## Data Availability

The data used and/or analyzed during the current study are available from the corresponding author upon reasonable request.
